# Hematological Parameters and Demographic Distribution of Hemoglobinopathies and Various Hemoglobin Variants

**DOI:** 10.7759/cureus.33115

**Published:** 2022-12-29

**Authors:** Israr A Shaikh, Rabia Zubair, Imran A Siddiqui, Asad H Ahmad, Umer Sheikh

**Affiliations:** 1 Pathology and Laboratory Medicine, Shaukat Khanum Memorial Cancer Hospital and Research Centre, Lahore, PAK; 2 Pathology, Shaukat Khanum Memorial Cancer Hospital and Research Centre, Lahore, PAK

**Keywords:** hemoglobin d, sickle cell disease: scd, β-thalassemia, fetal hemoglobin, hemoglobin: hb

## Abstract

Background

The study was conducted to find the prevalence of hemoglobinopathies along with their geographical/ethnic distribution to highlight the region of high prevalence that can be used to guide screening.

Method

Results of blood samples received for hemoglobin variants determination by high-performance liquid chromatography (HPLC) were retrospectively analyzed at Shaukat Khanum Memorial Cancer Hospital and Research Centre, Lahore. Blood samples were assayed for CBC (complete blood count), red blood cell morphology, and hemoglobin analysis by HPLC. CBC was performed on Sysmex XN 9000 analyzer (Sysmex, Kobe Japan), peripheral smears to review RBC morphology were stained with Wright-Giemsa stain, and HPLC was performed on BIO-RAD variant II (Bio-Rad Laboratories, Hercules, USA).

Results

Hemoglobinopathies were identified in 9.7% (n=997) out of 10,297 samples. Beta thalassemia trait was the most common hemoglobinopathy recognized with a prevalence of 5% (n=516), with the maximum number of cases in the Lahore district of Punjab province. The next most common hemoglobinopathy identified was sickle cell disease with a frequency of 1.43% (n=148) and the maximum cases from the Dera Ismail Khan district of the Khyber Pakhtunkhwa province. The additional important hemoglobinopathies found were sickle cell trait, hemoglobin-D Punjab trait, and compound heterozygote for sickle and beta thalassemia.

Conclusion

Hemoglobinopathies are the most common inherited disorders in Pakistan and worldwide. Screening for hemoglobinopathies is recommended in high-prevalence districts of Pakistan. Sickle cell screening is also recommended in newborns in the high prevalence area of Pakistan, such as the northwest regions.

## Introduction

Hemoglobinopathies are amongst the most common genetic illnesses around the world with an autosomal recessive pattern of inheritance [1]. Hemoglobinopathies are further categorized into two main groups: thalassemia syndromes and structural hemoglobin variants [2]. Thalassemia syndrome includes quantitative defects involving alpha and beta genes resulting in α- and β-thalassemia, respectively, requiring medical care such as regular blood transfusions [3]. Structural variants consist of hemoglobin S/Sickle cell (Hb-S), hemoglobin C (Hb-C), hemoglobin E (Hb-E) and hemoglobin D Punjab [4]. The incidence of beta thalassemia carriers is high in regions such as the Mediterranean, the Middle East, the Indian subcontinent, Southeast Asia, and South China [5]. With approximately 7% of the worldwide population being carriers of beta thalassemia gene mutation, hemoglobinopathies are one of the world’s major health problems [6].

The current international standard treatment for beta thalassemia syndrome includes hematopoietic stem cell transplantation (HSCT), which is the only curative treatment currently available [7]. Supportive treatment for thalassemia major includes lifelong regular transfusions combined with effective iron removal [8]. Hemosiderosis-related organ damage requires specific treatment [9, 10]. Sickle cell disease has specific complications such as pain crisis, acute chest syndrome, and cerebrovascular complications, which require specific management [11]. Screening and diagnosis of hemoglobinopathies require a comprehensive evaluation combining clinical and family history, blood counts, red blood cell indices, and molecular analysis [12]. Early diagnosis not only helps in genetic counselling but also plays an important role in the management and prevention of complications [13].

Screening requires robust methods and an accurate understanding of the prevalence of various hemoglobinopathies, which are currently lacking in developing countries, leading to an underestimate of the actual number of cases [14]. For a developing country like Pakistan, which is the home of approximately 221 million people [15], the disease burden of thalassemia and other hemoglobinopathies wreaks havoc on the healthcare system. Around 25% of the country’s blood donation deviates to patients suffering from various hemoglobinopathies [16], with a healthcare system that is not easily accessible to everyone in the country. Curative treatment such as hematopoietic stem cell transplantation (HSCT) is only affordable to a handful of patients, hence the need to raise more awareness and thorough screening process of thalassemia carriers throughout the country.

## Materials and methods

This is a descriptive cross-sectional study. We carried out a retrospective review of blood samples received for the determination of hemoglobin variants by high-performance liquid chromatography (HPLC) from January 2015 to December 2019 at Shaukat Khanum Memorial Cancer Hospital and Research Centre, Lahore. The architecture of the study was divided into the following steps: 1. Patient selection; 2. Sample for complete blood count collection and processing; 3. Red blood cell blood morphology evaluation; and 4. Determination and interpretation of various hemoglobin variants on high-performance liquid chromatography (HPLC).

Step 1: Patient selection

The laboratory at Shaukat Khanum Hospital receives blood samples for testing through collection centers all over Pakistan. However, around 99% of these samples are received from Punjab, Khyber Pakhtunkhwa (KPK), and Baluchistan provinces. These samples mostly include patients referred for testing by physicians based on history and clinical signs and symptoms.

Step 2: Sample for complete blood count collection and processing

**Figure 1:**
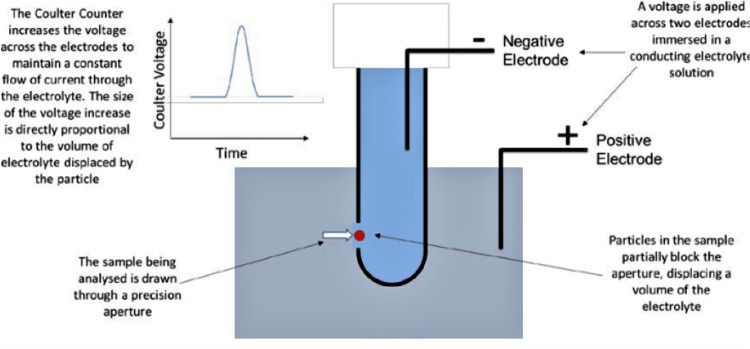
The Coulter counter principle Figure showing the Coulter principle schematic diagram for complete blood count analyzer which counts the number and size of the particle crossing the aperture as an electrode senses the difference in voltage. Figure is used with the permission of Beckman Coulter Life Sciences.

Whole blood was collected in a purple-top tube containing ethylene diamine tetra acetic acid (EDTA) as an anticoagulant. Blood samples were properly mixed immediately to prevent any clot formation. These blood samples were then transported at the temperature of 2-8°C within 24 hours. Blood samples received in the hematology section are checked for any clot with an applicator stick. If there were negative for clot, then they were sent to run on the XN-9000 CBC analyzer (Sysmex, Kobe, Japan), which detects the barcode and automatically aspirates the sample and sends the samples to respective sections for analysis. It works on the Coulter principle to count and measure the size of cells (Figure 1). Hemoglobin and red blood cell counts are measured directly while other parameters such as mean corpuscular volume (MCV), mean hemoglobin concentration (MCH), and hematocrit (HCT) are derived parameters. 

Step 3: Red blood cell blood morphology (RBC) evaluation

**Figure 2:**
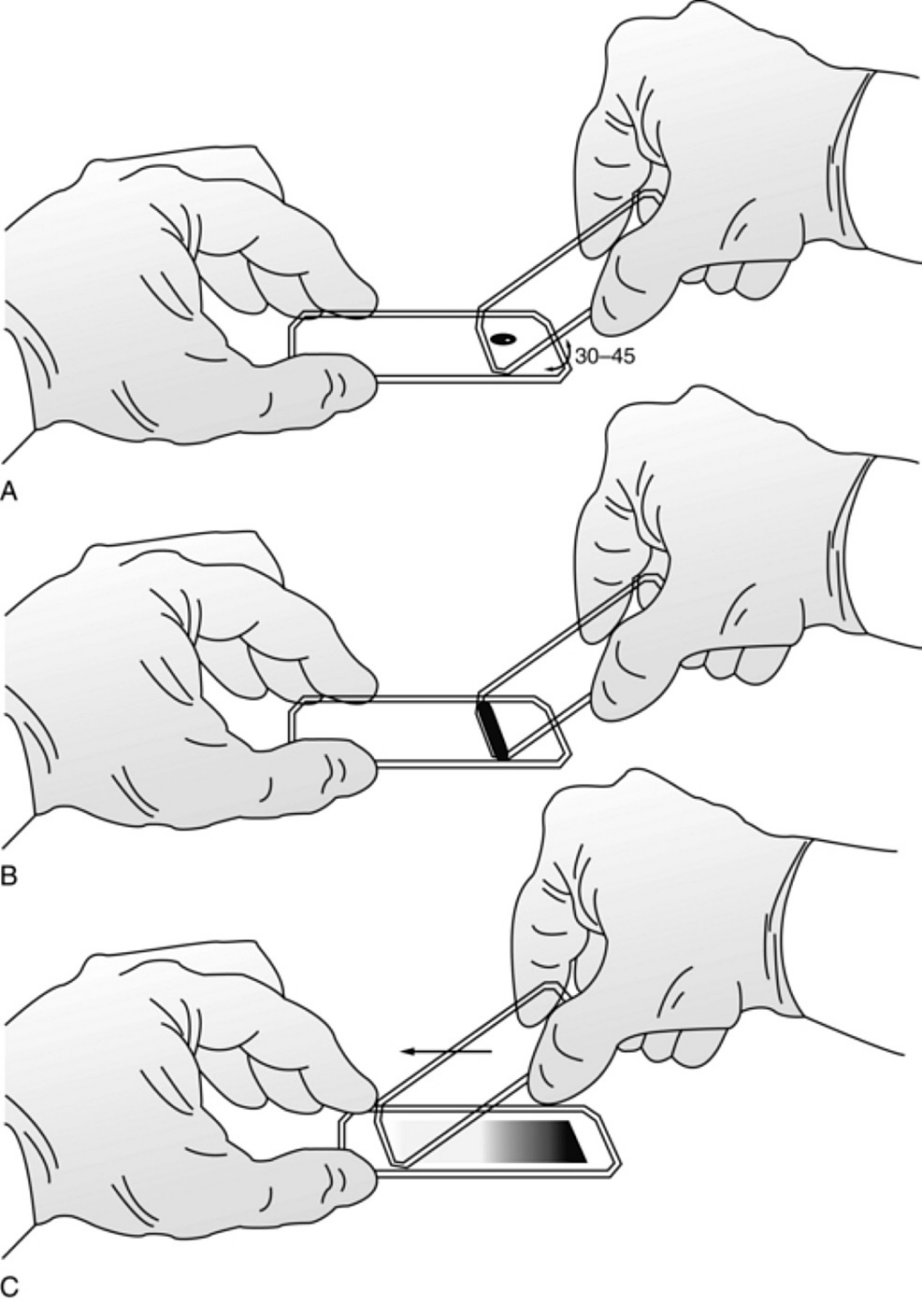
Method of preparing a peripheral smear Figure used with permission from Musculoskeletal Key (https://musculoskeletalkey.com/), .

Blood films were prepared by putting a small drop of blood on a glass slide with a dropper and then it was spread with another slide used as a spreader at an angle of 45 degrees. Caution was taken to spread the smear on the slide evenly and gently. Ideally, it should cover two-thirds of the slide (Figure 2). 

The smear was dried properly. Then it was fixed in methanol for 10-15 minutes. Diluted Giemsa stain was added for 25-30 minutes at room temperature. Then the slide was rinsed gently with running tap water. The slide was then air-dried and mounted with a coverslip and was ready for evaluation under the microscope. The slide was evaluated for red cell morphology and appropriate investigations were suggested based on age, counts on complete blood count (CBC), and red blood cell morphology.

Step 4: Determination and interpretation of various hemoglobin variants on high-performance liquid chromatography (HPLC)

**Figure 3:**
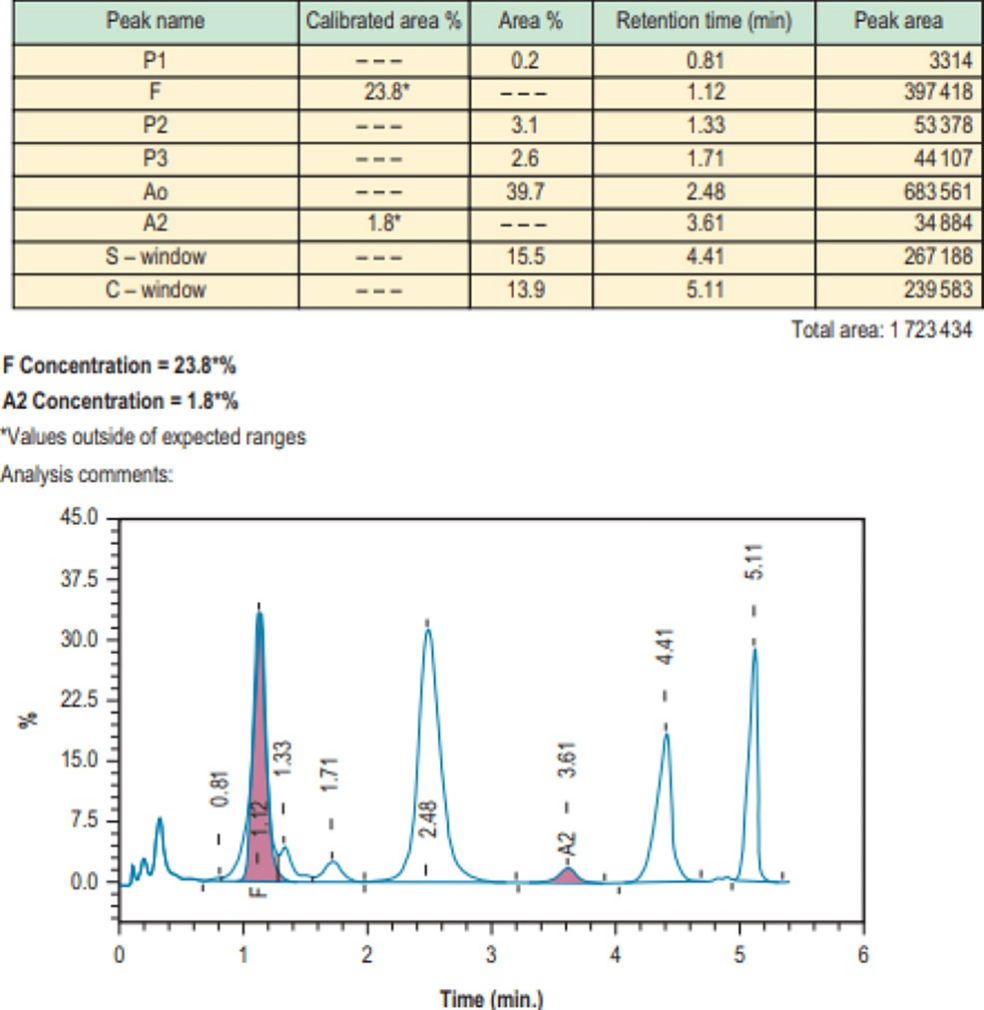
High-performance liquid chromatography Top: hemoglobin variant, percentage of specific hemoglobin, and area covered. Bottom: peaks of particular hemoglobin variant with respect to its retention time Figure is used with permission from *Dacie and Lewis Practical Hematology, 12th Edition *(Elsevier)

Cation-exchange HPLC separates many normal and variant Hb, including Hb A, A2, F, S, C, and D-Punjab. In HPLC, hemoglobin is adsorbed to a negatively charged resin column (stationary phase) and then eluted from the column by a positively charged solution that is added in increasing concentration and competes for binding to the negatively charged resin. Hemoglobin is thus differentially eluted at a rate related to its affinity for the column and detected by a photometer. The resulting elution patterns (Hb peaks) are graphically represented in defined "windows" based on their relative time to appearance in the eluate (retention time) (Figure 3). Retention times for different Hb fractions differ based on the HPLC equipment and software that is used; one should consult the manufacturer's specific reference for each instrument to identify Hb fractions based on retention times. The size of the Hb fractions can be quantified by computing the area under the curve for each peak.

The objective of our study was to determine (1) the frequency of various hemoglobinopathies; (2) the geographical distribution of various hemoglobinopathies in different regions of Pakistan; and (3) to set a goal that will help in targeted screening for a particular hemoglobinopathy with high prevalence in that region.

## Results

A total of 10,297 samples were assayed during our study period, out of which 9,272 were normal with no findings of hemoglobinopathy or any abnormal hemoglobin variant. Hemoglobinopathy/abnormal hemoglobin variant was present in 997 (9.7%) cases. Further breakdown of results according to specific haemoglobinopathy is given next.

Beta thalassemia trait

**Figure 4:**
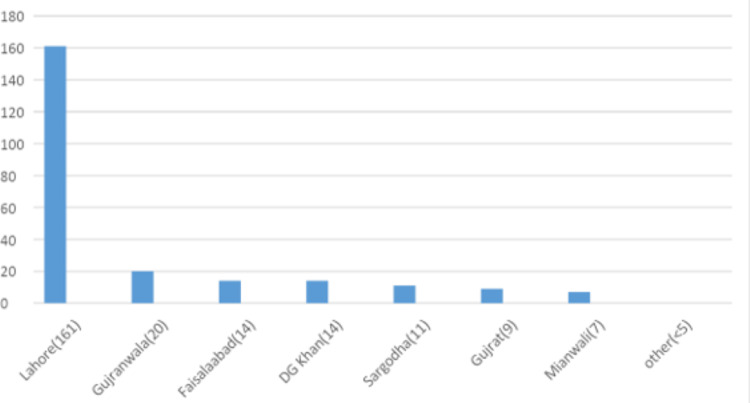
District-wise distribution of beta thalassemia trait in the Punjab province DG Khan: Dera Ghazi Khan

**Figure 5:**
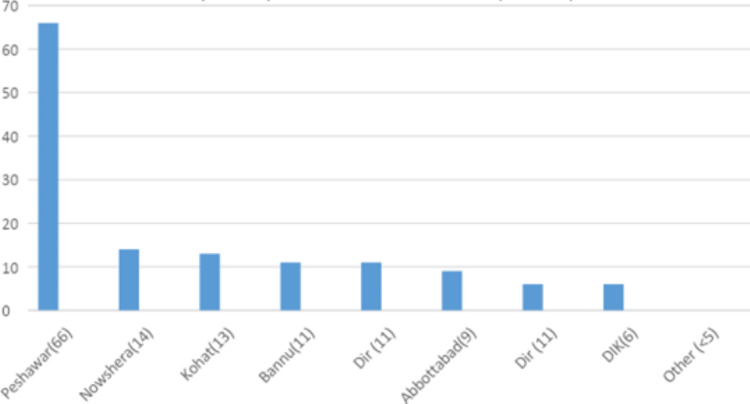
District-wise distribution of beta thalassemia trait in the Khyber Pakhtunkhwa (KPK) province DIK: Dera Ismail Khan

**Figure 6:**
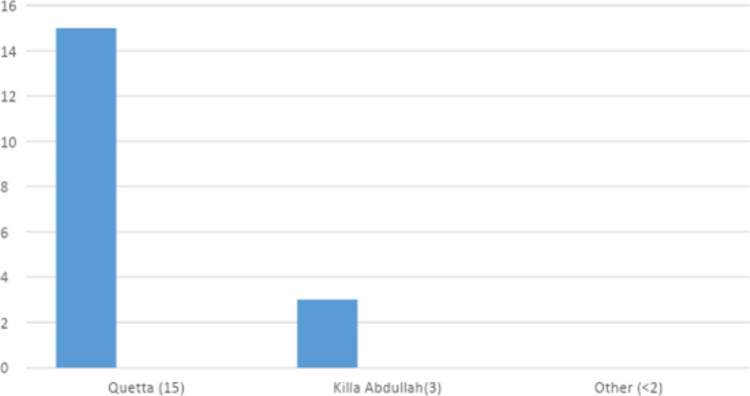
District-wise distribution of beta thalassemia trait in the Baluchistan province

Beta thalassemia trait was diagnosed in 516 (5%) patients. The mean age at diagnosis was 23.2 years. Almost all cases had microcytic hypochromic red blood cell indices based on MCV and MCH and elevated RBC counts (5.7 x1012/L). Mean hemoglobin A2 was 5.8 % and the mean Hb-F was 2% (Table 1). Punjab had the highest number of cases (n=301, 57.8%) (Table 2), with Lahore having most of the cases (Figures 4-6). 

**Table 1:** Age at diagnosis and hematological parameters of various hemoglobinopathies MCV: mean corpuscular volume; MCH: mean hemoglobin concentration; Hb: hemoglobin

	Age (years)	Hb (g/dl)	RBC X1012 /L	MCV (fl)	MCH (pg)	HbA%	HbA2%	HbF%	HbS%	HbD%
Beta Thalassemia Trait	23.2±14.6	10.5±2	5.7±1.5	60.2±5.4	18.6±2.1	91.8±5.8	5.8±0.7	2±1.8	0	0
Beta thalassemia syndrome	1.2±1	6.1±1.8	2.8±1	65.9±8.2	22±3.3	32±18	2.6±1.2	66.1±20	0	0
Sickle cell trait	20±15	11.8±2.7	4.7±0.9	75±9.8	24.9±4	61.4±6.7	2.7±0.6	5.2±2.8	31±8	0
Sickle cell disease	20±3	8.2±1.9	3±0.8	82±11.5	27±4.4	9.2±4	1.9±0.6	22.7±7.2	66±8.7	0
Sickle Beta Thalassemia	14.3±4.6	8±1.8	3.8±0.6	67.3±7.5	22±3.6	9±5	4.7±1	20.5±0.5	56±9.1	0
Hb-D Punjab trait	20.1±17.2	9.9±3.4	4.7±1.1	66.5±10	21.1±5.2	61.2±4.1	3±0.5	0	0	34.2±4
Hb-E trait	23±13	11±2.6	4.7±0.5	69.3±9.8	23±4.5	61±9	HbE- 37±9	2.3±1.8	0	0

**Table 2:** Province distribution of various hemoglobinopathies (frequency and percentage) Hb: hemoglobin

	Punjab	Khyber Pakhtunkhwa (KPK)	Baluchistan
Beta Thalassemia Trait	301 (57.8%)	188 (36.1%)	27 (5.2%)
Beta thalassemia syndrome	25 (64.1%)	8 (20.1%)	6 (15.4%)
Sickle cell trait	24 (26%)	41 (44%)	27 (30%)
Sickle cell disease	15 (10.13%)	78 (52.70%)	55 (37.16%)
Sickle Beta Thalassemia	4 (12.5%)	20 (62.5%)	8 (25%)
Hb-D Punjab trait	124 (79.5%)	21 (13.5%)	11 (7.1%)
Hb-E trait	11 (78.6%)	3 (21.4%)	0

Beta thalassemia syndrome

Beta thalassemia syndrome prevalence was found to be 0.37%. The mean age at diagnosis was 1.4 years and the mean Hb and Hb-F were 6.1 g/dl and 66.1%, respectively (Table 1). The majority of cases belonged to the Punjab province.

Sickle cell trait

The prevalence of the sickle cell trait was found to be 0.9%. The mean age at diagnosis was 20 years, while the mean Hb and sickle hemoglobin (Hb-S) were 11.8 g/dl and 31%, respectively, and the mean Hb-F was 5.2% (Table 1). 

**Figure 7:**
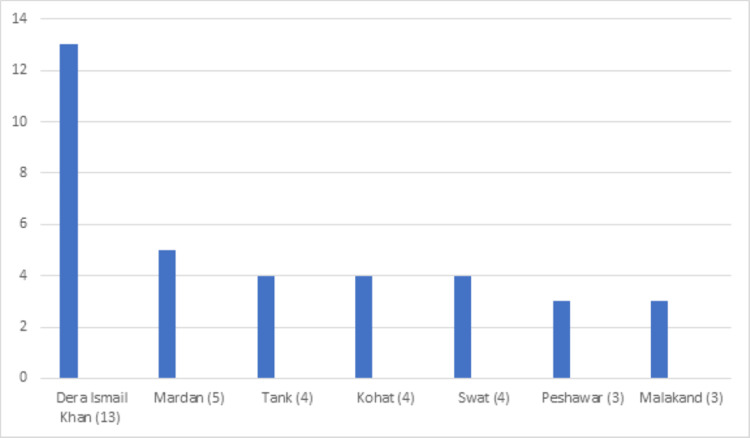
District-wise distribution of sickle cell trait in the Khyber Pakhtunkhuwa (KPK) province

**Figure 8:**
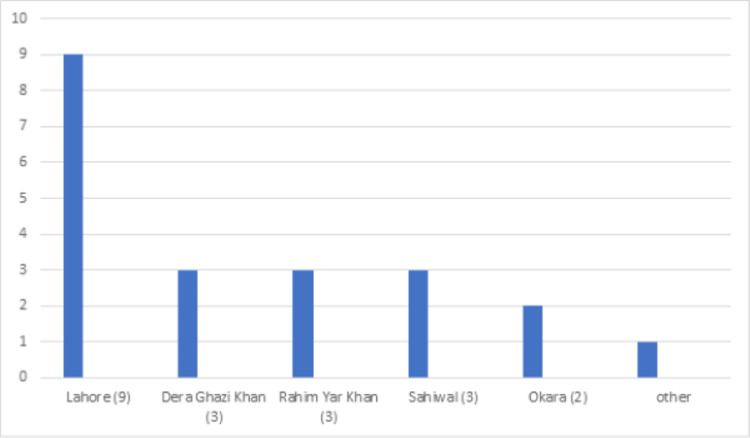
District-wise distribution of sickle cell trait in the Punjab province

**Figure 9:**
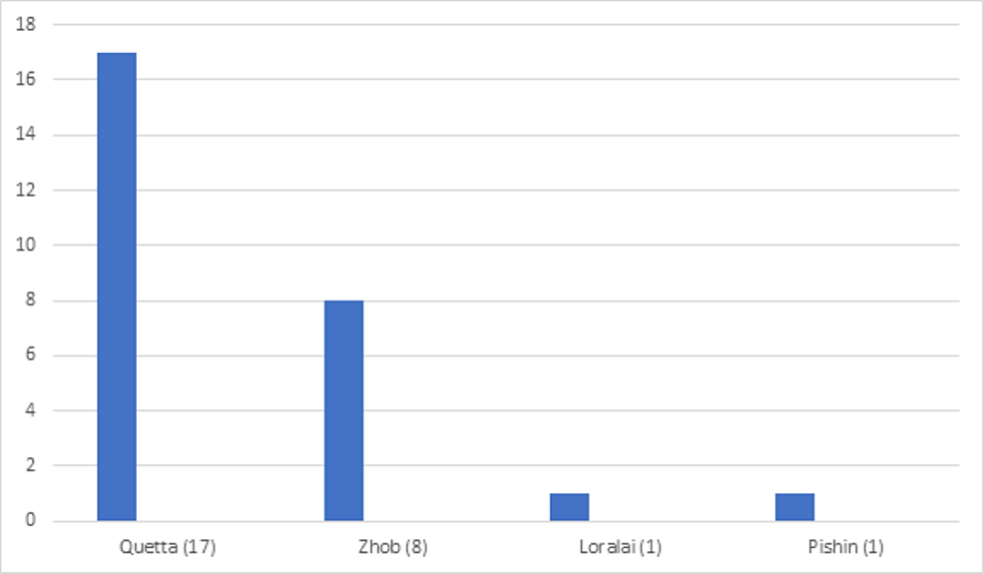
District-wise distribution of sickle cell trait in the Baluchistan province

Khyber Pakhtunkhwa (KPK) and Baluchistan (Table 2) had the most cases of sickle cell trait, with district Dera Ismail Khan in KPK and district Quetta in Baluchistan with the highest number of cases (Figures 7-9). 

Sickle cell disease

**Figure 10:**
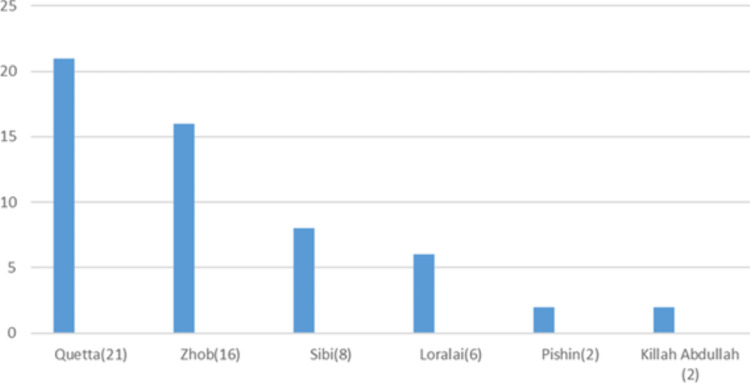
District-wise distribution of sickle cell disease in the Baluchistan province

**Figure 11:**
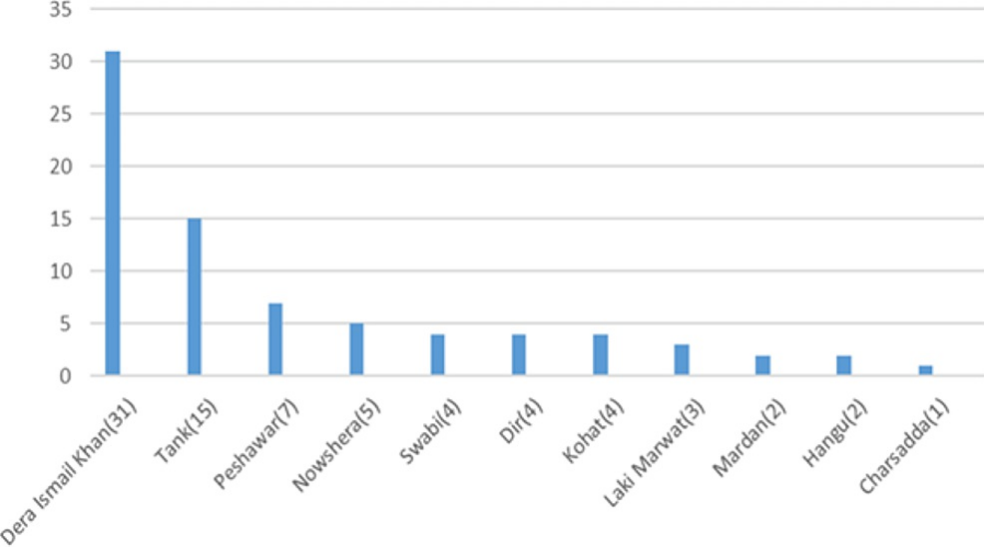
District-wise distribution of sickle cell disease in the Khyber Pakhtunkhwa (KPK) province

**Figure 12:**
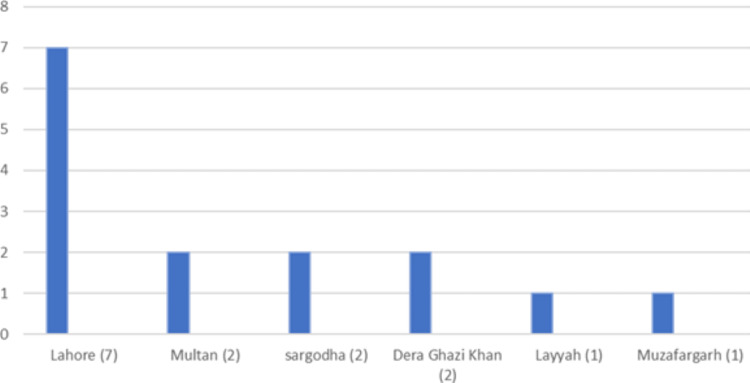
District-wise distribution of sickle cell disease in the Punjab province

Sickle cell disease was found in 148 (1.43 %) patients. The mean Hb was 8.2 g/dl while the mean Hb-S and Hb-F were 66% and 22.69%, respectively (Table 1). More than half of these patients (78, 52.70%) were from the Khyber Pakhtunkhwa province (Table 2). Further breakdown within the province showed district Dera Ismail Khan with the highest number of cases (31, 20.94%) (Figures 10-12). 

Sickle beta thalassemia 

The prevalence of compound heterozygotes for sickle beta-thalassemia was 0.31%, with a high prevalence in the Khyber-Pakhtunkhwa province (Table 2) and district Dera Ismail Khan with the highest number of patients. Mean Hb, HbA2, and Hb-S were 8.4 g/dl, 4.7%, and 56%, respectively (Table 1).

Hb-D Punjab trait

The prevalence of Hb-D Punjab trait was 1.5%. The mean age at diagnosis was 20.1 years, while the mean Hb and Hb-D percentage were found to be 9.9 g/dl and 34.2%, respectively (Table 1). Most cases were from the Punjab province (Table 2).

HbE trait

**Figure 13:**
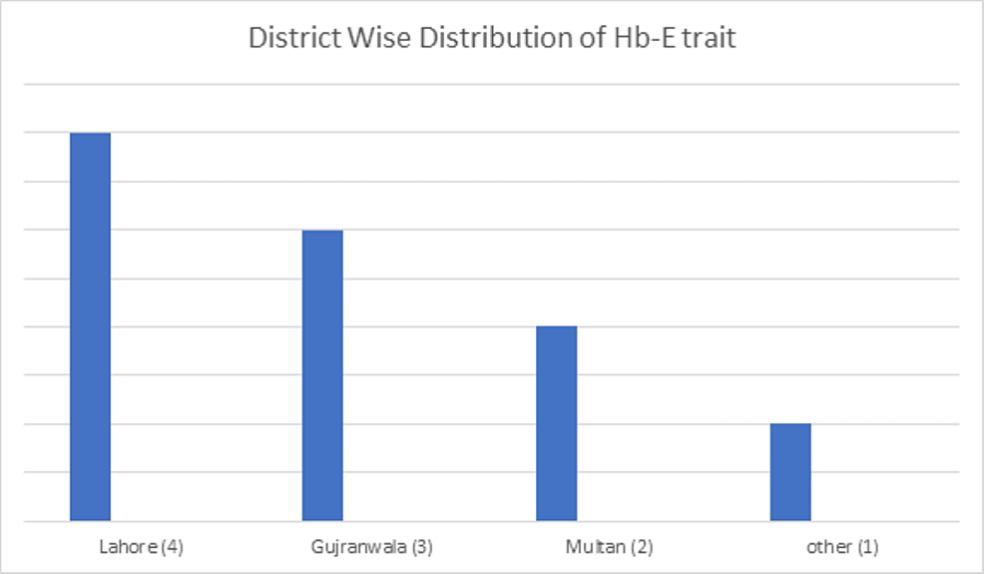
District-wise distribution of the Hb-E trait in the Punjab province

There were 14 cases of the Hb-E trait with a prevalence of 0.37%. The mean age at diagnosis was 23 years and the mean Hb-E percentage was 37% (Table 1) with most cases from the Punjab province (Table 2). Further district-wise distribution is shown in Figure 13.

## Discussion

The prevalence of hemoglobinopathies/abnormal hemoglobin variants was 9.7%, which is considerably lower than the study done by Shabbir et al. [17] in which the frequency was 34.2% in the most southern region of Pakistan; this might be related to selection bias of patients for hemoglobin variant analysis by high-performance liquid chromatography and the concurrent use of other methods for the detection of hemoglobin variants. While the combined prevalence in Bangladesh in a study [18] from South Asia was 5.2%. Beta thalassemia trait was the most common hemoglobinopathy identified with most cases in the region of Punjab, which is the southern part of Pakistan, and the Lahore district had the highest number of cases.

Sickle cell disease was the second most common finding of our study with a frequency of 1.43% (n=148), which is comparable to the result found in the study by Hashmi et al. [19], which was 1.92%. The Dera Ismail Khan district of the KPK province, which is in the northwest of the country, had the highest number of sickle cell disease patients with the Quetta district of Baluchistan province having the second-highest number of sickle cell disease patients. The prevalence of the sickle cell trait also followed a similar pattern to sickle cell disease with most cases from the Dera Ismail Khan district of Khyber Pakhtunkhwa and the Quetta district of Baluchistan province standing second in number. The mean age at diagnosis for sickle cell anemia was 20 years, which indicates that there is no screening program for sickle cell. Ideally, screening for sickle must be included in the newborn screening test for early diagnosis and counseling of parents regarding family planning, prevention of complications, and improve the quality of life. The mean hemoglobin F at diagnosis was 22.7±7.2, which was higher than what was found in African American patients, which is between 5% and 8%. This is the reason the complications of sickle cell disease are less prevalent in the South Asian population [20].

The frequency of compound heterozygous for beta thalassemia trait and sickle cell trait was 0.31 % (n=28). Most of the cases were found in the regions where sickle cell trait was with high prevalence, such as the Dera Ismail Khan district of Khyber Pakhtunkhwa and the Quetta district of Baluchistan. 

The lower incidence of sickle cell trait and compound heterozygote for sickle cell trait and beta thalassemia trait than sickle cell disease is unusual. This may be because these samples are submitted by patients on request from the physician and are not part of a population-based screening program. Sickle cell disease is more likely to have a significant clinical presentation, so this selection bias may have resulted in higher incidence in our study. 

The limitation of our study is that it is not a general screening program; rather, available data was retrospectively analyzed. So it may not reflect the actual number and distribution of hemoglobinopathies.

## Conclusions

Hemoglobinopathies are the most common inherited disorders in Pakistan and worldwide. Screening for hemoglobinopathies is recommended in infants after 6 months of age in high-prevalence districts of Pakistan for particular hemoglobinopathy. Sickle cell screening is also recommended in newborns in the high prevalence area of Pakistan, such as the northwest regions of Pakistan. Government and private sector can play a significant role by targeting these high-prevalence areas in raising awareness about the importance of screening to reduce the disease burden.
